# Trait and state anxiety are mapped differently in the human brain

**DOI:** 10.1038/s41598-020-68008-z

**Published:** 2020-07-06

**Authors:** Francesca Saviola, Edoardo Pappaianni, Alessia Monti, Alessandro Grecucci, Jorge Jovicich, Nicola De Pisapia

**Affiliations:** 1 Center for Mind/Brain Sciences (CIMeC), University of Trento, Rovereto, TN Italy; 2 Department of Psychology and Cognitive Sciences (DiPSCo), University of Trento, Corso Bettini 31, 38068 Rovereto, TN Italy; 3 Department of Neurorehabilitation Sciences, Casa Di Cura Privata del Policlinico, Milan, Italy

**Keywords:** Psychology, Cognitive neuroscience, Emotion

## Abstract

Anxiety is a mental state characterized by an intense sense of tension, worry or apprehension, relative to something adverse that might happen in the future. Researchers differentiate aspects of anxiety into state and trait, respectively defined as a more transient reaction to an adverse situation, and as a more stable personality attribute in experiencing events. It is yet unclear whether brain structural and functional features may distinguish these aspects of anxiety. To study this, we assessed 42 healthy participants with the State-Trait Anxiety Inventory and then investigated with MRI to characterize structural grey matter covariance and resting-state functional connectivity (rs-FC). We found several differences in the structural–functional patterns across anxiety types: (1) trait anxiety was associated to both structural covariance of Default Mode Network (DMN), with an increase in dorsal nodes and a decrease in its ventral part, and to rs-FC of DMN within frontal regions; (2) state anxiety, instead, was widely related to rs-FC of Salience Network and of DMN, specifically in its ventral nodes, but not associated with any structural pattern. In conclusion, our study provides evidence of a neuroanatomical and functional distinction between state and trait anxiety. These neural features may be additional markers in future studies evaluating early diagnosis or treatment effects.

## Introduction

The human anxious psychophysiological response was first scientifically described by Sigmund Freud as a feeling of imminent and pressing danger that could be based on objective or moral risk^1^. A more contemporary definition^2,3^ considers anxiety as a mental state characterized by an intense sense of tension, worry or apprehension, relative to something adverse that might happen in the future. Anxiety can be an adaptive response driving coping behaviours to face possible dangers, but if excessive and unmotivated, can become dysfunctional, paving the way for developing anxiety disorders.

One core issue in the field has been to distinguish between anxiety as “*state anxiety*” defined as a temporary reaction to adverse events, and “*trait anxiety*”, a more stable personality feature^4^, defined as a constant individual difference related to a tendency to respond with concerns, troubles and worries to various situations. *Trait anxiety* is thought to belong to a list of characteristic traits of an individual’s personality^5^, and it can be associated with different psychopathological conditions and constant high arousal. Conversely, *state anxiety* is a more transient intense emotional state, associated with a temporary increased sympathetic nervous system activity^5^, but with no specific pathological conditions. Nonetheless, whether the two anxiety types are behaviourally correlated, or independent features, still remains unanswered. According to Spielberg’s^6^ early formulation, anxiety is a unidimensional construct including both *state* and *trait anxiety*, considered to be different sides of the same coin. In this theoretical frame, the anxious individual is characterized by a personality trait combined with a predisposition to an increased phasic anxiety level in dangerous or stressful situations. However, other authors suggested *trait* and *state anxiety* to be separate multidimensional constructs^7,8^.

With the aim of better understanding their neural correlates, these two anxiety types have been investigated in healthy populations, both jointly and as separate constructs, using a variety of neuroimaging techniques. Concerning structural grey matter (GM), *trait anxiety* is related to volume alterations in limbic regions, such as amygdala, parahippocampal gyrus, inferior temporal gyrus and inferior frontal cortex^9^ and abnormal cortical thickness in amygdala and cingulate regions^10^. At the functional level, *trait anxiety* impacts anterior cingulate cortex and medial prefrontal cortex activity during decision-making tasks^11^and mediates, in these regions, a compensatory response in cognitively demanding tasks^12^. As far as *state anxiety* is concerned, neuroimaging studies focusing on this aspect are lacking. Reported structural GM modification in *state anxiety* are missing and functional changes are mainly recognized as the result of anxious feelings induced during the MRI scanning^13,14^. Considering both anxiety types, the neural limbic system is thought to play a prominent role; in particular, amygdala activation is believed to be mediated by anxiety in unconscious emotional vigilance^15^. Indeed, the amygdala does not show a typical suppression in response to threat in *state anxiety* when the attentional focus is engaged in other tasks^16^, but people with high *trait anxiety* were found to be prone to distraction in the presence of emotional stimuli^17^. A clearer separation of anxiety types, and a better understanding of their neural bases, could be relevant for the clinical practice, especially if one considers that *trait anxiety* is a risk factor for mood and anxiety disorders^18,19^. Evidence exists of the fact that high *trait anxiety* individuals are vulnerable to develop stress-induced depression or anxiety disorders, because they display hyper-responsivity to stressful situations, increased passive coping responses to environmental challenges, alterations in cognitive functions, and lower social competitiveness^18^. Altogether, these factors facilitate the development of psychopathological disorders making the investigation of neural correlates of the two anxiety types of crucial importance.

Indeed, anxiety disorders are thought to be the outcome of limbic system disturbances^20^. Limbic regions such as amygdala and cingulate cortices are both functionally and structurally involved in anxiety disorders^21,22^, consequentially inducing an imbalance in the brain’s emotional centres^20^, which presumably drives the anxious symptomatology. Furthermore, anxiety disorders are frequently investigated as a matter of connectivity changes: several findings reported functional changes and highlighted an increased functioning of the cingulo-opercular network, also known as Salience Network (SN)^23,24^, and decreased functional connectivity (FC) in the Default Mode Network (DMN) while performing emotion regulation tasks^25–27^. In this framework, Sylvester et al., 2012, proposed a new functional network model of anxiety that considers the anxious behavioural phenomenon and anxiety disorders as a combination of disturbances in brain FC^28–31^. Authors suggested a characteristic FC pattern for anxiety, where an increased functioning in the SN^12,32,33^ is associated with a decrease in the regulation exploited by the DMN^22,25,26,34–36^. The stated dissociation between these two main large-scale functional networks is reported to be at the basis of *trait anxiety* and anxiety disorders, potentially useful in differentiating this diagnostic entity from other mental disorders. Even so, this model does not account for such connectivity disruptions to be present in *state anxiety*. Indeed, the functional networks dysfunction is presented only considering the personality anxious trait and the resulting pathological anxiety disorder, without elucidating what happens when the two types of anxiety are considered separately. Moreover, it is still not clear to which extent anxiety is represented by these changes in FC patterns and how these patterns are distinguished in the healthy population between the transient emotional state of anxiety and the more stable and premorbid *trait anxiety*^37–39^.

Taken together, all these studies support the idea that anxiety is a complex phenomenon where both structural and functional changes are taking place, but to the best of authors’ knowledge, none of these has tried to investigate *trait* and *state anxiety* simultaneously, both at brain structural and functional networking levels. Furthermore, the extent to which these changes happen, and how they interact with the two types of anxiety, is still not well defined. This creates a need for deepening the understanding of both anxiety types to picture a larger framework where *state* and *trait anxiety* are described both in terms of differences and communalities.

In this study, our aim is to characterize neural during rest while awake that can distinguish between *trait* and *state anxiety*, both at structural and functional brain levels. With our data-driven techniques we expected to find that the two anxiety types will be reflected differently at the neural level, in particular with *trait anxiety* impacting on structural GM and *state anxiety* resulting in perturbations of FC patterns. Particularly, *trait anxiety*, being a more stable individual difference encoded in the personality, should affect GM of fronto-temporal areas including the cingulate gyrus for their role in emotion processing and the top-down control of subcortical areas. Whereas, *state anxiety*, for its transient nature, should affect more the FC level, especially the SN, for its role in detecting potentially dangerous stimuli, and the DMN, for its function in emotion regulation, rumination and worry. Furthermore, we hypothesize that the functional dissociative pattern between DMN and SN described in Sylvester et al. 2012 applies to variations in *trait* and *state anxiety* in a healthy population.

## Results

Gender differences in age and anxiety tests results were controlled across all participants and none of them proved to be relevant (Age (t(40) = 0.5301, *p* = 0.5989); STAI-Y1_STATE_ (t(40) = 0.6900, *p* = 0.4942); STAI-Y2_TRAIT_ (t(40) = 0.2093, *p* = 0.8353). The STAI-Y1_STATE_ scores had a mean of 31 ± 4.9 and a range of 21–44; STAI-Y2_TRAIT_ scores had a mean of 41.1 ± 9.7 with a range of 27–71. The STAI-Y1_STATE_ scores did not significantly correlate with STAI-Y2_TRAIT_ scores (r = 0.2516, *p* = 0.1080), while still showing a positive association.

### Trait anxiety

#### Source-based morphometry

The multivariate SBM analysis was performed on 42 participants and returned 20 ICs. SBM results were then correlated with *trait anxiety* scores (STAI-Y2 _TRAIT_) using Pearson’s parametric correlation. The Sources are numbered in terms of relevance of anatomical structures in *trait anxiety* by means of previous evidence.

**Figure 1:**
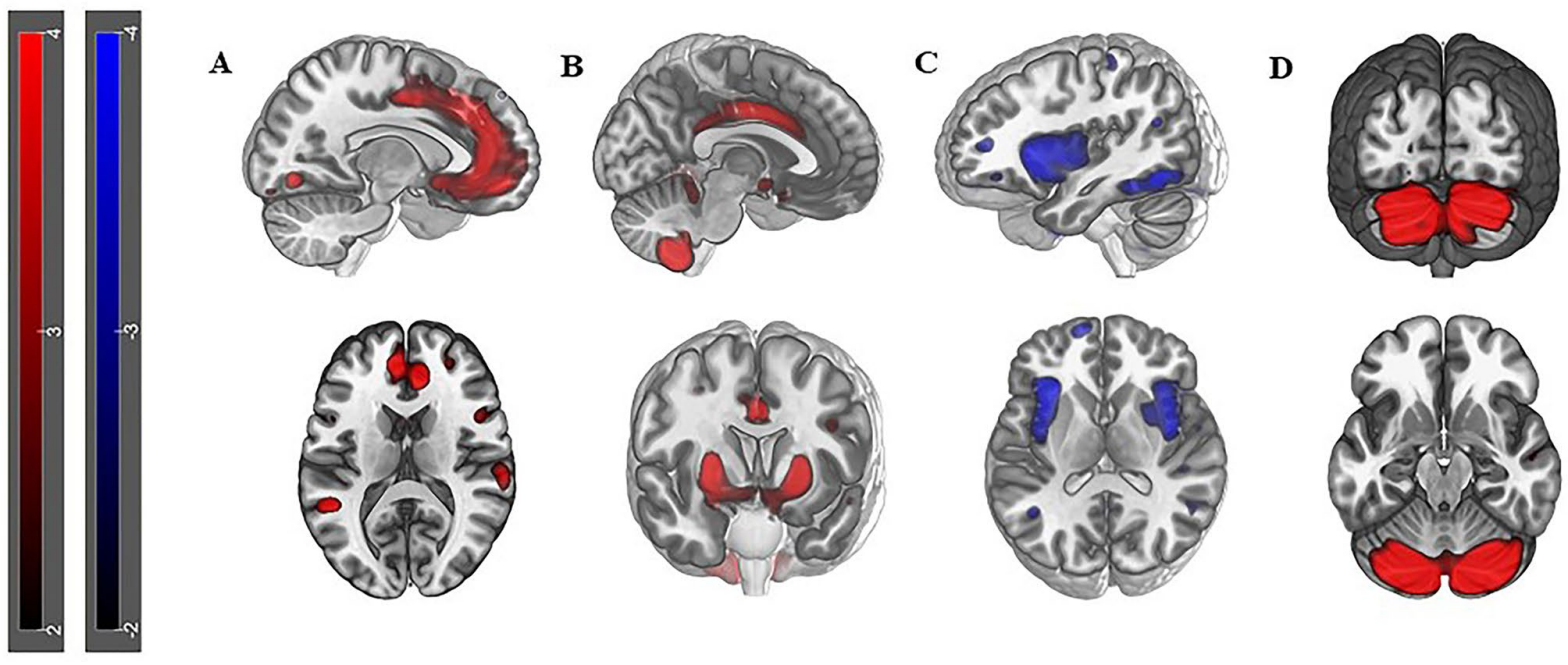
Surface reconstruction of Sources: **A** Reconstruction of Source 1 (r = 0.3, *p* value = 0.04) in the sagittal view exhibiting positive structural covariance in the anterior cingulate; **B** Reconstruction of Source 2 (r = 0.4, *p* value = 0.03) in sagittal and axial view showing positive structural covariance in limbic regions such as amygdala and cingulate gyrus; **C** Reconstruction of Source 3 (r =  − 0.5, *p* value = 0.001) in the sagittal view, showing negative spatial pattern of covariance, in both hemispheres, mostly located in precuneus, cuneus and inferior frontal gyrus; **D** Source 4 (r = 0.5, *p* value = 0.0004) highlighting a strong positive structural covariance cerebellar involvement.

**Figure 2:**
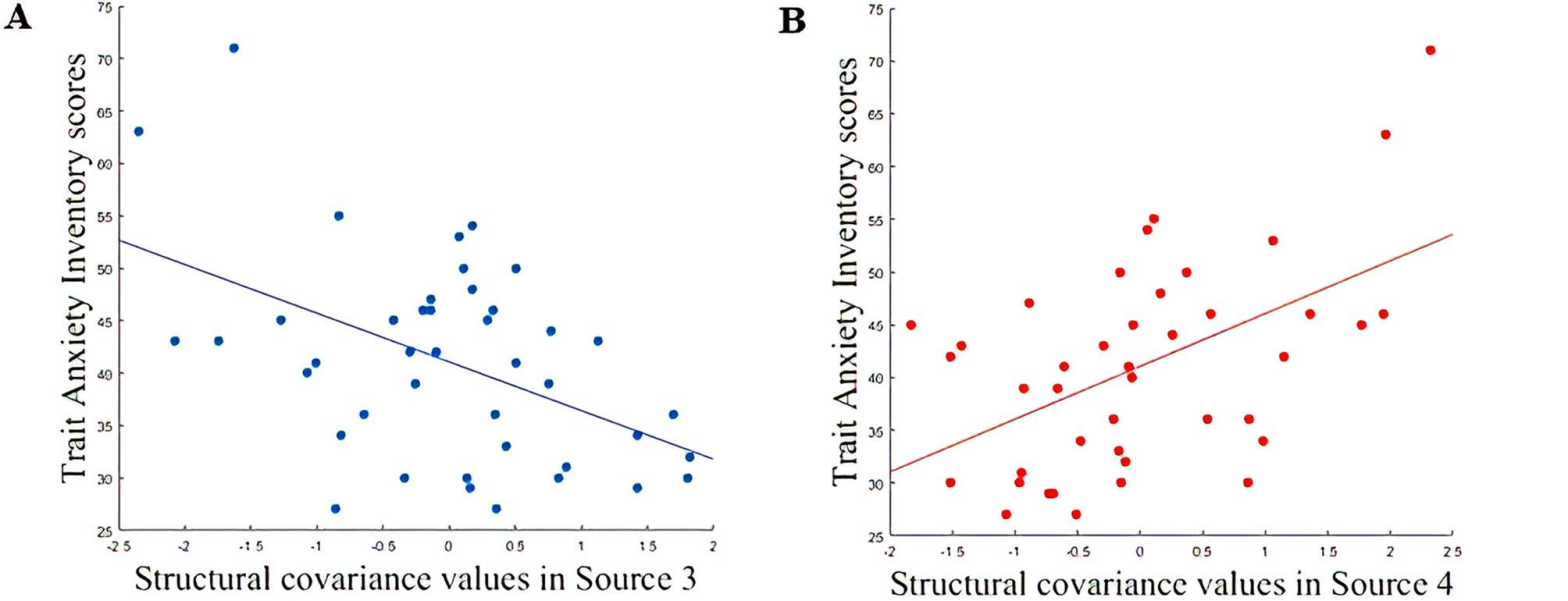
**A** Correlation plot of Source 4 and STAI-Y2_TRAIT_ scores (r = 0.5, *p* value = 0.0004; *p* value_FDR_ = 0.007). The caption is showing a positive correlation with the structural covariance network mainly localized in cerebellar areas. **B** Correlation plot of Source 3 and STAI-Y2_TRAIT_ scores (r =  − 0.5, *p* value = 0.001; *p* value_FDR_ = 0.009). The caption is showing a negative correlation with the structural covariance network mainly localized in precuneus, cuneus and middle temporal gyrus.

Four Sources resulted to be significantly correlated with *trait anxiety* (*p* values < 0.05): (1) positive correlation with STAI-Y2_TRAIT_ scores in Source 1 including limbic structures as cingulate gyrus (Fig. 1A, Table 1, r = 0.3, *p* value = 0.04), Source 2 including temporal and frontal region (Fig. 1B, Table 1, r = 0.4, *p* value = 0.03) and Source 4 including portions of the cerebellum (Fig. 1D, Table 1, r = 0.5, *p* value = 0.0004); (2) negative correlation with STAI-Y2_TRAIT_ scores in Source 3 including portions of the precuneus, the cuneus and of the middle temporal gyrus (Fig. 1C, Table 1, r =  − 0.5, *p* value = 0.001). When the FDR correction is applied for testing the different 20 Sources, only Source 4 (*p* value_FDR_ = 0.0076) and Source 3 (*p* value_FDR_ = 0.0095) resulted to survive. Source 4 and 3 correlations, which are the most explicative in terms of statistic power and strength of correlation, are showed in Fig. 2. Specific details about significant Sources compositions and locations are described in Table 1.

**Table 1:** Demographic information of participants including age, gender, educational years and STAI-Y assessment.

	Participants	
Gender (M/F)	24/19	
	Mean	SD
Age (years)	23.8	2.19
Education (years)	15.4	11.17
STAI-Y1 state anxiety	31.0	11.45
STAI-Y2 trait anxiety	41.1	9.45

#### Functional connectivity analysis

**Figure 3:**
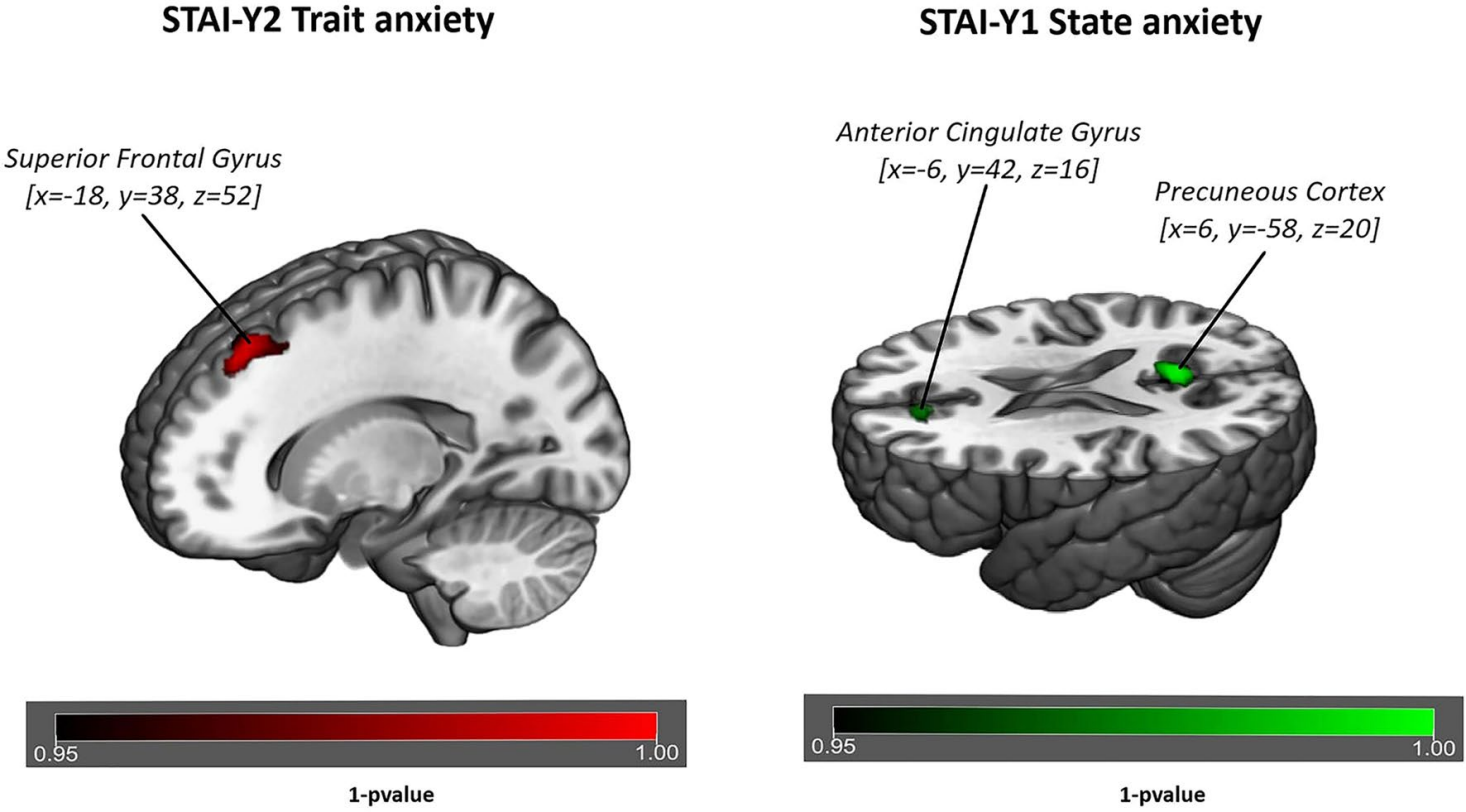
Surface rendering of functional connectivity changes in the Default Mode Network related to trait anxiety (left of figure) and state anxiety (right of figure) respectively. Trait anxiety is shown to be correlated to functional connectivity of the Default Mode Network in the superior frontal gyrus (*p* value = 0.007) for *p* < 0.05 threshold corrected for multiple comparisons (TFCE) across voxels and *p* < 0.012 for Bonferroni correction across different components. State anxiety is shown to be correlated to functional connectivity of the Default Mode Network in the precuneus (*p* value = 0.003) and in anterior cingulate (*p* value = 0.0016) for *p* < 0.05 threshold corrected for multiple comparisons (TFCE) across voxels and *p* < 0.012 for Bonferroni correction across different components.

A significant correlation with *trait anxiety* (STAI-Y2 _TRAIT_), was detected in FC of the IC identified as the DMN^40^. *Trait anxiety* is shown to be associated with increase FC of the DMN in frontal regions such as superior-frontal gyrus and middle frontal gyrus (*p* value = 0.007, Fig. 3, Table 2). Conversely the SN is not showing any FC correlation with *trait anxiety*.

**Table 2:** Anatomical labelling for statistically significant Sources correlated with trait anxiety.

Source	*p* value	r-value	MNI peak coordinates	Anatomical labelling of the Harvard–Oxford atlas
Source 1	0.04	0.3	[− 6, 34, 30]; [12, 41, 13]	Middle Frontal Gyrus
			[− 50, − 39, 5]	Middle Temporal Gyrus
			[− 3, 36, 24]; [9, 42, 1]	Anterior Cingulate
			[− 3, 33, 27]; [9, 22, 29]	Cingulate gyrus
			[− 50, − 46, 11]; [64, − 24, 11]	Superior Temporal Gyrus
Source 2	0.03	0.4	[− 24, 3, 4]; [25, 2, 5]	Lentiform Nucleus
			[59, − 37, − 11]	Middle Temporal Gyrus
			[− 1, − 54, − 38]; [3, − 53, − 41]	Cerebellar Tonsil
			[− 3, − 59, − 41]; [3, − 59, 41]	Inferior Semi-lunar Lobule
			[− 27, − 62, 35]; [31, − 61, 36]	Precuneus
			[0, − 1, 33]; [3, − 4, 32]	Cingulate Gyrus
			[− 45, − 37, 39]; [34, − 61, 39]	Inferior Parietal Lobule
Source 3	0.001^a^	− 0.5	[− 49, − 38, 28]	Inferior Parietal Lobule
			[− 42, − 59, 15]; [15, − 61, 38]	Precuneus
			[43, − 66, 25]	Middle Temporal Gyrus
			[− 52, − 38, 31]	Supramarginal Gyrus
			[53, − 17, 30]	Postcentral Gyrus
			[− 16, − 11, 61]; [36,1,44]	Middle Frontal Gyrus
			[− 16, − 71,10]	Cuneus
Source 4	0.0004^a^	0.5	[− 18, − 71, 10]; [16, − 81, − 19]	Declive
			[− 13, − 80, − 23]; [16, − 80, − 23]	Uvula
			[− 24, − 80, − 29]; [28, − 74, − 28]	Tuber
			[24, − 74, − 27]	Pyramis
			[− 24, − 85, − 18]; [25, − 75, − 14]	Fusiform Gyrus
			[− 28, − 61, − 23]; [31, − 61, − 23]	Culmen
			[− 28, − 75, − 37]; [13, − 71, − 37]	Inferior Semi-lunar Lobule

The table is showing the strength of correlation (Pearson’s correlation coefficient), the MNI Coordinates of the peak, the anatomical name of the area. ^a^Sources surviving FDR correction for multiple comparison across 20 ICs.

### State anxiety

#### Source-based morphometry analysis

The multivariate SBM analysis was performed on 42 participants and returned 20 ICs, as set in the analysis features. SBM results were then correlated with *state anxiety* scores (STAI-Y1 _STATE_) using Pearson’s parametric correlation. None of the 20 Sources returned by SBM were found to be significantly correlated with STAI-Y1 _STATE_ (all *p* values > 0.05).

#### Functional connectivity analysis

**Figure 4:**
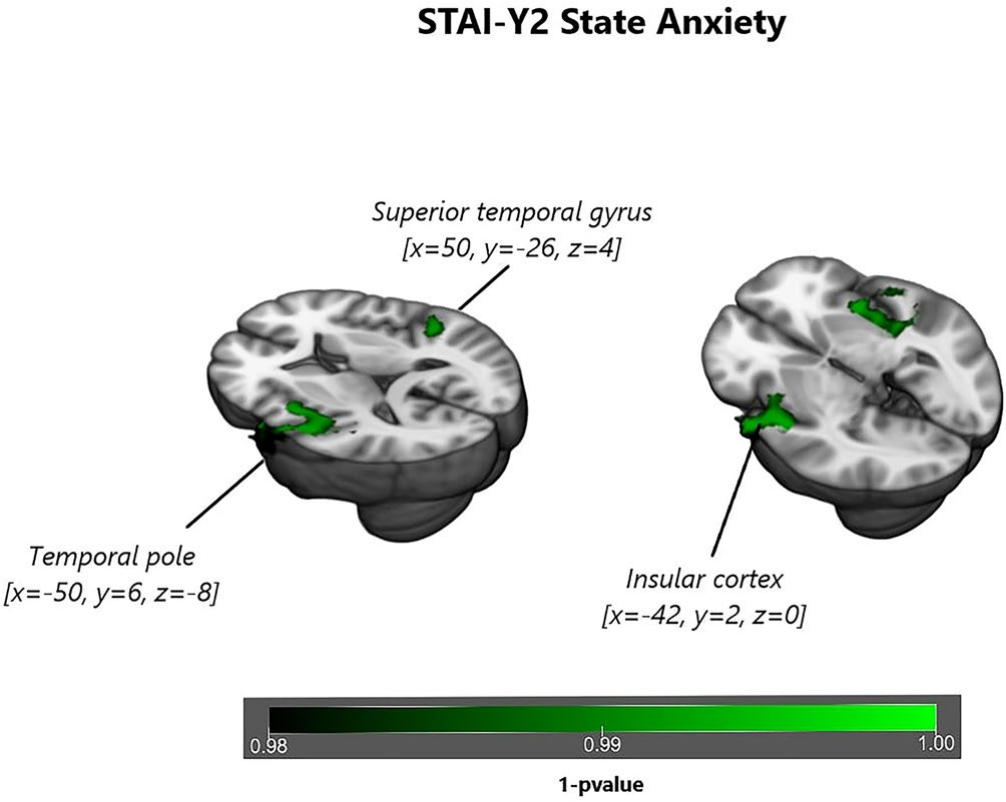
Surface rendering of functional connectivity changes in the Salience Network related to State anxiety. State anxiety is shown to be correlated to functional connectivity of the Default Mode Network in the temporal pole (*p* value = 0.007), superior temporal gyrus (*p* value = 0.006) and in insular cortex (*p* value = 0.008) for *p* < 0.05 threshold corrected for multiple comparisons (TFCE) across voxels and *p* < 0.012 for Bonferroni correction across different components.

Significant correlation with *state anxiety*, measured with STAI-Y1 _STATE_, was detected in FC of the ICs identified as DMN and SN^40^. *State anxiety* is shown to be associated with: (1) an increased FC of the DMN in posterior/ventral regions such as precuneus and posterior cingulate (*p* value = 0.003, Fig. 3, Table 2) and (2) an increased FC of the SN in temporal regions such as precentral gyrus, planum polare and Insula (*p* value = 0.007, Fig. 4, Table 3).

**Table 3:** Clusters of reliable voxels for changes in resting-state functional connectivity in the Default Mode Network associated respectively with Trait (upper rows) and State anxiety (lower rows).

STAI-Y	*p* value	Cluster index	MNI peak coordinates	Anatomical labelling of the Harvard–Oxford atlas
Trait anxiety	0.007	1	[− 18, 38, 52]	Superior Frontal Gyrus, Frontal Pole, Middle Frontal Gyrus
	0.029	1	[− 18, 22, 52]	Superior Frontal Gyrus, Middle Frontal Gyrus
State anxiety	0.003	2	[6, − 58, 20]	Precuneous Cortex, Cingulate gyrus, posterior division, Supracalcarine Cortex
	0.035	2	[10, − 46, 32]	Intracalcarine cortex, Cuneal Cortex
	0.036	2	[− 10, − 58, 16]	Cingulate Gyrus, posterior division, Precuneous Cortex
	0.016	1	[− 6, 42, 16]	Precuneous Cortex, Supracalcarine Cortex, Intracalcarine Cortex
	0.026	1	[− 6, 38, − 4]	Cingulate Gyrus, posterior division
	0.03	1	[− 2, 38, 4]	Paracingulate Gyrus, Cingulate gyrus, anterior division
	0.035	1	[− 2, 26, 12]	Cingulate Gyrus, anterior division, Paracingulate Gyrus, Frontal Medial Cortex

MNI Coordinates of the peak, peak labelling and for *p* < 0.05 threshold corrected for multiple comparisons (TFCE) across voxels are reported for each cluster.

## Discussion

The aim of this study was to investigate the neural basis of the trait/state distinction of anxiety in a group of healthy volunteers. One hypothesis for this distinction is that the more stable and personality aspects (trait) are implemented in structural configurations, whereas the temporary aspects of anxiety (state) correlate with functional patterns of brain activity during awake rest with eyes closed. Our main findings support this view, as we found that, while the correlation of *state and trait anxiety* does not reach significance in our sample of participants, *trait anxiety* correlates with GM structural covariance of DMN and SN nodes and with the FC of the DMN, whereas *state anxiety* correlates with the FC of DMN and SN.

Therefore, our results will be discussed in terms of how the DMN and SN, both with reference to structure and function, are represented and contribute to the double construct of anxiety, e.g. trait and state. When referring to anxiety, the DMN and the SN can be seen as two sides of the same coin: one side is related to self-generated thoughts, rumination, mind-wandering as the more inner component of anxiety, whereas the other side is associated to salience processing, cognitive control as the behavioural modality of facing stimuli/stressors.

Starting from the DMN, we found that its structural GM nodes covary positively with *trait anxiety* in fronto-temporal regions (e.g. medial frontal cortex, medial/superior temporal cortex) and negatively in parietal one (e.g. precuneus, cuneus, inferior parietal lobule); conversely, *state anxiety* is not structurally related with any GM structures. Previous findings highlighted how fronto-temporal nodes of the DMN are involved in social cognition and emotion processing^41,42^. Additionally prefrontal cortex, while being related to anxiety, showed structural GM changes^43^ which are thought to indirectly mediate aberrant functioning of the amygdala circuitry^44,45^. Indeed, since frontal regions are key components of emotion regulation systems^46^, and considering *trait anxiety* as a stable and enduring characteristic of the individual, it is plausible that these structurally altered regions mediate improper connections with the amygdala circuitry, which are instead intact in the transient emotional state.

Furthermore, we reported that functionally the DMN was frontally associated with *trait anxiety*, while precuneus cortex was correlated with *state anxiety*. This functional dissociation appears to embrace the double role of DMN functional nodes, where (1) on one hand, the prefrontal cortex has a prominent role in regulating/supressing anxiety feelings and exploiting executive control^47^, (2) and on the other, the posterior cingulate/precuneal regions are involved in adapting behaviour to environmental changes and attentional control^48,49^. That could explain why if the individual is experiencing *state anxiety* the brain transiently changes functional connections serving attentional processing and generates maladaptive behaviour by means of making hyper-relevant emotional events. While in a reciprocal way, the functional network is durably disturbed in *trait anxiety*, since the structural nodes are altered and can no longer sub-serve efficiently functional connections within the emotion regulation system. The observed effect may be established due to GM density anomalies in specific regions (e.g. frontal DMN), which by modifying the statistical dependency of the GM hemodynamic response in that defined areas, potentially give rise to abnormal FC. Nevertheless, this relationship is not one to one and we cannot assume that for a defined structural change a related FC alteration will be observed in so far as: (1) FC per se does not always depend on structural correlates but can be influenced by it^50,51^; and (2) functional abnormalities, in this study, are also extended to other brain regions where, due to the transient nature of the *state anxiety*, there are no apparent structural abnormalities. Nevertheless, consequently, in *trait anxiety*, emotionally relevant stimuli are always treated as salient, and the absence of regulation makes this condition a risk factor for anxiety disorder^18,19,52^.

This framework easily connects to our results within the SN: anterior cingulate cortex was positively correlated with *trait anxiety* while considering its GM structure, whereas it was functionally positively associated with *state anxiety*. The SN, by detecting salient changes in the surrounding environment, broadly connects the cingulate cortex and the insula with the emotion relay node of the amygdala^53^, which was found to be functionally associated with *state anxiety* and structurally with *trait anxiety*^54,55^. For this reason, our findings expand experimentally the model proposed by Sylvester et al., by confirming the involvement of both DMN and SN in anxiety, but at the same time highlight how the double construct of anxiety is represented differently at the neural level in respect to which anxiety type is considered. We did not replicate the dissociative pattern between the two networks^56^, but we described how different nodes of the two networks are structurally and functionally connected in different fashions based on the type of anxiety. This may allow a potential improvement in the study of therapeutic intervention for anxiety disorders. Indeed, starting from the strong structural involvement of the DMN nodes in *trait anxiety*, we can better associate the “*neuroticism*” to the putative biomarker of excessive rumination^57^ characterized by a hyper-functioning of the frontal part of DMN, which disrupts FC with the amygdala. At the same time, we can better describe *state anxiety* as temporary anxious avoidance behaviour^57^, where attention circuits comprising the precuneus are disrupted in favour of SN hyper-connectivity.

In this picture, by combining behavioural exhibitions and neural correlates, it will be easier to recognise the perdurable condition of an anxious personality trait from transient worries, and establish suitable approaches to regulate it (e.g. with TMS, Cognitive-behavioural therapy or Mindfulness). According to this, a strategic improvement in anxiety regulation in high *trait anxiety* individuals could be achieved via pharmacological and/or neurostimulation methods (e.g. TMS, or tDCS, over nodes of SN/DMN) targeting the specific areas found in this study. Such treatments could be used in anxiety disorders, in a preventive way in subclinical populations, as well as for improving diagnostic procedures by including specific neuroimaging biomarkers. Finally, these findings may lead to the creation of new diagnostic tools and treatments based on neuroscientific findings aimed at ameliorating symptoms of anxiety disorders.

However, there are some limitations in this study, which can restrict the generalizability of our results. Firstly, our sample is relatively small, and even if recent findings demonstrate no agreement in finding a biomarker for anxiety with larger sample size in favour of a stronger cognitive hypothesis^58^, future studies could try to replicate our results using distinct and/or larger datasets. Moreover, while considering the two types of anxiety (e.g. trait and state), we used only one psychometric test to measure it. A more comprehensive analysis could include multiple assessments to introduce several levels of description of the anxious phenomena, to reduce potential biases inherent to particular tests. Furthermore, *state anxiety* was measured subsequently after the MRI scan, thus introducing some limitations in correlating the psychometric score with functional activity of the brain during the MRI. Further studies should address this issue by adding complementary information of *state anxiety* of the participant during scanning, for example, by measuring sympathetic response or arousal levels by means of galvanic skin response, heart rate, and respiratory rhythms.

From a methodological point of view, our functional analysis was based on static functional connectivity. This approach assumes that resting-state or intrinsic networks are stationary during data acquisition. However, evidence suggests that networks fluctuate, as studied in the emerging field of functional dynamic connectivity. Various analytical methods have been proposed to estimate dynamic properties of functional connectivity, including sliding-window, time–frequency, point-process, temporal graph analyses, as described in various reviews^59^. The comparison of dynamic metrics across these methods is still a matter of research^60^, making the interpretation of results from studies challenging. In future studies, these methods could be of interest to characterize temporal fluctuations of connectivity, particularly if anxiety fluctuations could be also monitored or manipulated independently^61^. By means of dynamic functional connectivity, one could improve knowledge about how brain networks interact and are activated in precise moments during scanning. In this framework, the functional correlation of precuneal regions with *state anxiety* could be better associated with the exact timing of the adapting behavioural response in the scanner and understood as a function of its coupling or anti-coupling with the dynamics of the SN. Concerning *trait anxiety*, the potentially disrupted regulation function of DMN frontal regions could be observed in relationships to dynamics of the anterior cingulate cortex (here structurally related to trait anxiety), which, as subpart of the SN, could have time-varying properties different from the whole SN. Therefore, dynamic functional connectivity could bring to further insights about how FC patterns of the DMN and SN are paired in association to the double anxiety construct.

Summarizing, in this original study we attempt to decouple the double anxiety construct^62^ and show, by comparing its structural and functional neural correlates, how *trait* and *state anxiety* are mapped differently in the healthy human brain. Further studies could corroborate these findings by directly measuring anxiety levels during scanning and looking at the synchronous dynamic changes in FC related to *trait* and *state anxiety*.

## Materials and methods

### Participants

Forty-two healthy participants took part in this study (19 females and 23 males, mean age = 23.8 years, sd = 4.4, age range 19–38 years). This study was approved by the “Ethics Committee on experiments involving human beings” of the University of Trento, and all research was performed in accordance with relevant guidelines and regulations. All participants signed a written informed consent, according to guidelines set by the Ethics Committee. The inclusion criteria to participate in this study were to be aged between 18 and 45 years; absence of a history of psychiatric or neurological disease, not currently on psychoactive medications and no contraindication to MRI environment (claustrophobia, ferromagnetic material in the body, etc.). A detailed description of demographical information of participants is listed in Table 4. Participants first underwent a structural and functional Magnetic Resonance Imaging (MRI) acquisition (details below) and then answered to the State-Trait Anxiety Inventory (STAI-Y)^4^ at the end of the neuroimaging session. The STAI-Y consists of 40 self-report items related to anxiety, for both state (STAI-Y1_STATE_) and trait (STAI-Y2_TRAIT_) components, adapted and standardized for the Italian population^4,63^. STAI-Y items are rated on a 4-point Likert scale. The range for each subtest (STAI-Y1_STATE_ and STAI-Y2_TRAIT_) is 20–80, a higher score indicating greater anxiety. The behavioural data were analysed with MATLAB R2016b: we tested for any a-priori gender, STAI-Y1 _STATE_ and STAI-Y2_TRAIT_ differences and correlation between STAI-Y1 _STATE_ and STAI-Y2_TRAIT_ in our sample.

**Table 4:** Clusters of reliable voxels for changes in resting-state functional connectivity in the Salience Network associated with State anxiety (lower row).

STAI-Y	*p* value	Cluster index	MNI peak coordinates	Anatomical labelling of the Harvard–Oxford atlas
State anxiety	0.007	3	[− 50, 6, − 8]	Precentral Gyrus, Inferior Frontal Gyrus, pars opercularis, Central Opercular Cortex
	0.007	3	[− 46, − 6, − 8]	Planum Polare, Heschl's Gyrus, Insular Cortex, Frontal Operculum Cortex
	0.007	3	[− 38, 6, 0]	Insular Cortex, Central Opercular Cortex
	0.007	3	[− 62, − 2, − 12]	Precentral Gyrus, Postcentral Gyrus, Central Opercular Cortex
	0.008	3	[− 54, − 18, 8]	Heschl's Gyrus, Planum Temporale, Central Opercular Cortex, Planum Polare
	0.009	3	[− 58, − 10, 24]	Postcentral Gyrus, Precentral Gyrus
	0.006	2	[50, − 26, 4]	Superior Temporal Gyrus, posterior division, Planum Temporale
	0.007	2	[38, − 18, − 8]	Insular Cortex, Heschl's Gyrus, Planum Polare
	0.007	2	[38, 10, − 4]	Insular Cortex
	0.009	2	[38, − 6, 0]	Insular Cortex
	0.013	2	[50, − 10, 4]	Planum Polare, Heschl's Gyrus, Superior Temporal Gyrus
	0.013	2	[58, − 2, − 4]	Superior Temporal Gyrus, anterior division, Planum Polare, Heschl's Gyrus
	0.048	1	[− 42, − 14, 40]	Precentral Gyrus, Postcentral Gyrus

MNI Coordinates of the peak, peak labelling and for *p* < 0.05 threshold corrected for multiple comparisons (TFCE) across voxels are reported for each cluster.

### Image acquisition

Imaging data were acquired using a 4 T Bruker MedSpec Biospin MR scanner with a birdcage transmit and 8-channel receive head radiofrequency coil. For the resting-state functional connectivity (rs-fMRI) acquisition participants were instructed to lay still, with their eyes closed and to think of nothing in particular. The scanning duration of the rs-fMRI was approximately 8 min (215 volumes). Rs-fMRI images were acquired with a single shot T2*-weighted gradient-recalled echo-planar imaging (EPI) sequence (TR = 2,200 ms, voxel resolution = 3 × 3 × 3  mm^3^, TE = 33 ms, FA = 75°, FOV = 192 × 192  mm^2^; slice gap, 0.4 mm).

Moreover, a structural T1- weighted anatomical scan was acquired (MP-RAGE; 1 × 1 × 1  mm^3^; FOV, 256 × 224 mm^2^; 176 slices; GRAPPA acquisition with an acceleration factor of 2; TR, 2,700 ms; TE, 4.18 ms; inversion time (TI), 1,020 ms; 7° flip angle).

### Image pre-processing

Off-line quality assurance (QA) was performed, before and after pre-processing steps, to check for the quality of data and to detect gross distortion or artefacts; after this step no image was discarded. QA prior to pre-processing included visual inspection of the acquired images, tSNR and standard deviation maps inspection; whereas after pre-processing, motion parameters and power spectra of the rs-fMRI time series were examined together with co-registration and normalization steps to ensure the correct progress of data analysis. Structural T1-weigthed images were pre-processed with SPM12 software (https://www.fil.ion.ucl.ac.uk/spm/soiware) through the Computational Anatomy Toolbox 12 (CAT12) (https://www.neuro.uni-jena.de/cat/). During this process, for structural images, the origin was set to reorient the image, then the T1-weighted images were segmented in GM, white matter (WM) and cerebro-spinal fluid (CSF). As in Ashburner and Friston^64^, alignment and normalization to MNI space were performed with smoothing application of full width at half maximum of Gaussian smoothing kernel [8, 8, 8]. Resting-state data were analysed with FMRIB Software Library (FSL^65^). Images Pre-processing steps include: (1) reorientation of the volumes and head motion correction performed with the rigid body transformation default settings and motion outliers regression; (2) slice timing correction; (3) brain extraction for both EPI motion corrected sequence and T1-weighted image; (4) spatial smoothing using a 6 mm full-width half maximum Gaussian kernel (twice the voxel size of the images^66^); (5) temporal high-pass linear filtering (100 s cut-off); (6) and finally co-registration and normalization to standard MNI template with final resampling of the functional image to 4 mm.

### Source-based morphometry

Source Based Morphometry (SBM) is a multivariate whole-brain approach based on Independent Component Analysis (ICA)^67^. ICA application to MRI images can recognize maximally spatially independent sources in order to reveal pattern of covariation between participants that characterize T1 anatomical images. This provides information of GM structural covariance among participants. SBM was performed using the GIFT software (https://trendscenter.org/software/gift/). The software was set to detect 20 Independent Components (ICs) by default as the best option for low model order studies^67–70^ Infomax algorithm was chosen to compute the analysis, and the ICASSO (https://research.ics.aalto.fi/ica/icasso/) was chosen to investigate the reliability of ICA. Both RandInit and Bootstrap modes were selected in ICASSO analysis setup, and ICA was run 100 times with a minimum cluster size set up to 80. The main goal of SBM is to find a numerical vector that displays the GM volume of each component for each participant. Once the analysis has been run, SBM creates a matrix where columns refer to the sources and rows refer to participants. This matrix indicates how a specific IC is expressed in each subject. A parametric correlation test was then used to test for correlations in all subjects between each subject’s matrix and STAI-Y1_STATE_ and STAI-Y2_TRAIT_ anxiety scores. All results are reported both at *p* < 0.05 uncorrected and then at *p* < 0.05 FDR corrected^71,72^ for multiple comparison across the 20 ICs.

### Resting-state functional connectivity analysis

The pre-processed resting-state fMRI data was then analysed using Multivariate Exploratory Linear Optimized Decomposition into Independent Components 3.0 (MELODIC). The multiple 4D data sets were decomposed into their distinct spatial and temporal source components using ICA, which is the same methodology used by SBM in terms of detecting maximally independent cortical networks of GM variations. As the aim of the analysis was to detect group association of resting-state FC changes associated with anxiety, we did not assume consistent temporal responses within subjects. Therefore, the ICA group analysis was temporarily concatenated (FSL^65^). The ICs number was manually set to 20^73^ as low-order model analysis. In order to separate noise components from the underlying resting-state networks, ICs were tested for their correlation (threshold of r-value > 0.2) to labelled networks^40^. Subsequently, 8 ICs out of 20 were identified as noise (r-value < 0.2) and were discarded from the analysis (Table S1). As a final step in the network identification, ICs was visually inspected by expert users to detect consistency between ICs matching with template networks (high correlation values)^40,74^ resemble well-known functional networks (see Supplementary materials). Subsequently, we performed a dual regression to investigate group differences in resting-state networks related to STAI-Y. To do so, based on our a-priori hypotheses, a one sample t-test, randomised with permutation testing, was performed on 2 ICs (see Supplementary materials, Figure S1) respectively belonging to the DMN (r-value = 0.57) and SN^40^(r-value = 0.40) to detect correlations in *state* and *trait anxiety*, measured with the State-Trait Anxiety Inventory (STAI-Y), demeaned across subjects before the testing as the main covariate of interest. Finally, statistical correlations were tested using non-parametric permutation testing, with threshold-free cluster enhancement (TFCE). This was done to depict specific FC patterns correlated to the two anxiety types. Resting-state FC results are reported at *p* value = 0.012, for *p* < 0.05 threshold corrected for TFCE multiple comparisons across voxels, and *p* < 0.012 threshold with Bonferroni correction for multiple comparisons across the 2 IC for the 2 scores of STAI-Y1_STATE_ and STAI-Y2_TRAIT_.

## Supplementary information

Supplementary file1

## Data Availability

The datasets generated during and/or analysed during the current study are not publicly available but are available from the corresponding author on reasonable request.
