# Supplementary material for: Trait and state anxiety are mapped differently in the human brain
Source: Sci Rep. 2020 Jul 6;10:11112. doi: 10.1038/s41598-020-68008-z (PMC7338355; doi:10.1038/s41598-020-68008-z)
Supplement: Supplementary file 1 — Supplementary file1 [file 41598_2020_68008_MOESM1_ESM.docx]

**Supplementary materials**

**Trait and state anxiety are mapped differently in the human brain**

Francesca Saviola^1^, Edoardo Pappaianni^2^, Alessia Monti^3^, Alessandro Grecucci^2^, Jorge Jovicich^1^, Nicola De Pisapia^2 *^

1. CIMeC, Center for Mind/Brain Sciences, University of Trento, Rovereto (Trento), Italy
2. DipSCo, Department of Psychology and Cognitive Sciences, University of Trento, Rovereto (Trento), Italy
3. Department of Neurorehabilitation Sciences, Casa di Cura Privata del Policlinico, Milan, Italy

*For correspondence:

Nicola De Pisapia, Department of Psychology and Cognitive Science (DipSCo)

University of Trento, Corso Bettini 31, 38068, Rovereto (TN), Italy

Cell. ++39 346 5437734; Tel. ++39 0464 80 8787

Email: [nicola.depisapia@unitn.it](mailto:nicola.depisapia@unitn.it)

Materials and Methods

**Independent Components selection**

Eight Independent Components (IC) out of 20 did not satisfy the criteria of cross-correlation with the atlas template (Yeo et al., 2011), were subsequently identified as noise (r-value<0.2) and discarded from the analysis (Table S1).

Based on the a-priori hypothesis of the study, we aimed to identify the Default mode Network (DMN) and the Salience Network (SN), which by looking at the matching with template networks (high correlation values) were recognized as IC 8 and 12 (SN) and IC 5 and 16 (DMN). As a further indicator of robustness and strength of the functional connections in the IC, mean and range of the z-score value were investigated (e.g. representing the strength of connection between the average voxels belonging to the IC and a defined voxel within the IC) resulting in: IC 5 (range [0-9.87], mean 1.3); IC 8 (range [0-11.52], mean 1.7); IC 12 (range [0-8.92], mean 1.6); IC 16 (range [0-8.51], mean 0.7). Moreover, explained variance by each network was taken into account in the IC component selection, with IC 5 (5.5 %) and IC 8 (5.1%) describing higher percentage of the dataset compared to IC 12 (4.7 %) and IC 16 (4.3 %). Finally, visual inspection of the components was performed to detect the consistency of resemble of well-known functional networks.

Subsequently, IC 5 (DMN) and IC 8(SN) were selected as the IC of interest in the main analysis since the highest z-score values and percentage of variance explained combined with a high correlation value with the network template.

For consistency, IC 12 (SN) and IC 16 (DMN) were tested for the same hypothesis and report coherent results showing significant effect for STAI-Y1_STATE_ only (Table S2 and Table S3). Cluster peaks are consistent with results reported in the main manuscript especially in Insular Cortex and Temporal Pole for the Salience Network STAI-Y1_STATE_  effect. On the other hand, Default mode Network STAI-Y1_STATE_  effect in this case involves Inferior-Medial Temporal regions.

| **Functional Networks** | **Atlas Volume** | **IC** | **r-value** |
| --- | --- | --- | --- |
| *Visual Network* | 1 | 1 | 0.465 |
|  | 1 | 2 | 0.527 |
|  | 1 | 4 | 0.209 |
|  | 1 | 9 | 0.25 |
| *Somatosensory Network* | 2 | 6 | 0.596 |
|  | 2 | 8 | 0.447 |
| *Dorsal attention Network* | 3 | 6 | 0.248 |
|  | 3 | 9 | 0.226 |
|  | 3 | 10 | 0.389 |
| *Ventral attention Network* | 4 | 8 | 0.4 |
|  | 4 | 12 | 0.414 |
| *Limbic Network* | 5 | 11 | 0.384 |
| *Fronto-parietal Network* | 6 | 10 | 0.414 |
|  | 6 | 12 | 0.301 |
|  | 6 | 15 | 0.434 |
| *Default mode Network* | 7 | 5 | 0.573 |
|  | 7 | 16 | 0.428 |
|  |  |  |  |

Table S1. **Functional network consistency with the reference atlas (Yeo et al., 2011).** Correlation values between the ICs and the reference atlas networks from Yeo functional networks. Only IC with a r-value>0.2 are considered functionally relevant, whereas other ICs are discarded as noise. Based on our a-priori hypothesis we select IC 5 as the Default mode Network and IC 8 as the Salience Network (which is comprehend on the Ventral Attention Network).

| **STAI-Y** | **p-value** | **Cluster index** | **MNI peak coordinates** | **Anatomical labelling of the Harvard-Oxford atlas** |
| --- | --- | --- | --- | --- |
| **STATE ANXIETY** | 0.019  0.033  0.049 | 3  2  1 | [54, 10, -32]  [-58, -2, -36]  [62, 18, -16] | Temporal Pole, Middle Temporal Gyrus  Middle Temporal Gyrus, Inferior Temporal Gyrus  Temporal Pole |

**Table S2.** Clusters of reliable voxels for changes in resting-state functional connectivity in the Default mode Network associated with State anxiety. MNI Coordinates of the peak, peak labelling and for **p < 0.05** threshold corrected for multiple comparisons (TFCE) across voxels are reported for each cluster.

| **STAI-Y** | **p-value** | **Cluster index** | **MNI peak coordinates** | **Anatomical labelling of the Harvard-Oxford atlas** |
| --- | --- | --- | --- | --- |
| **STATE ANXIETY** | 0.002  0.013  0.028  0.036  0.039  0.048 | 6  5  4  3  2  1 | [-6, 14, 36]  [-42, 6, 0]  [46, 18, -16]  [-18, -10, 4]  [22, 14, 8]  [34, 38, 28] | Anterior Cingulate Gyrus, Paracingulate Gyrus  Insular Cortex, Central Opercular Cortex  Temporal Pole, Fronto-orbital Cortex  Pallidum, Thalamus  Putamen, Caudate  Frontal Pole, Inferior Frontal Gyrus, pars triangularis |

**Table S3.** Clusters of reliable voxels for changes in resting-state functional connectivity in the Salience Network associated with State anxiety. MNI Coordinates of the peak, peak labelling and for **p < 0.05** threshold corrected for multiple comparisons (TFCE) across voxels are reported for each cluster.


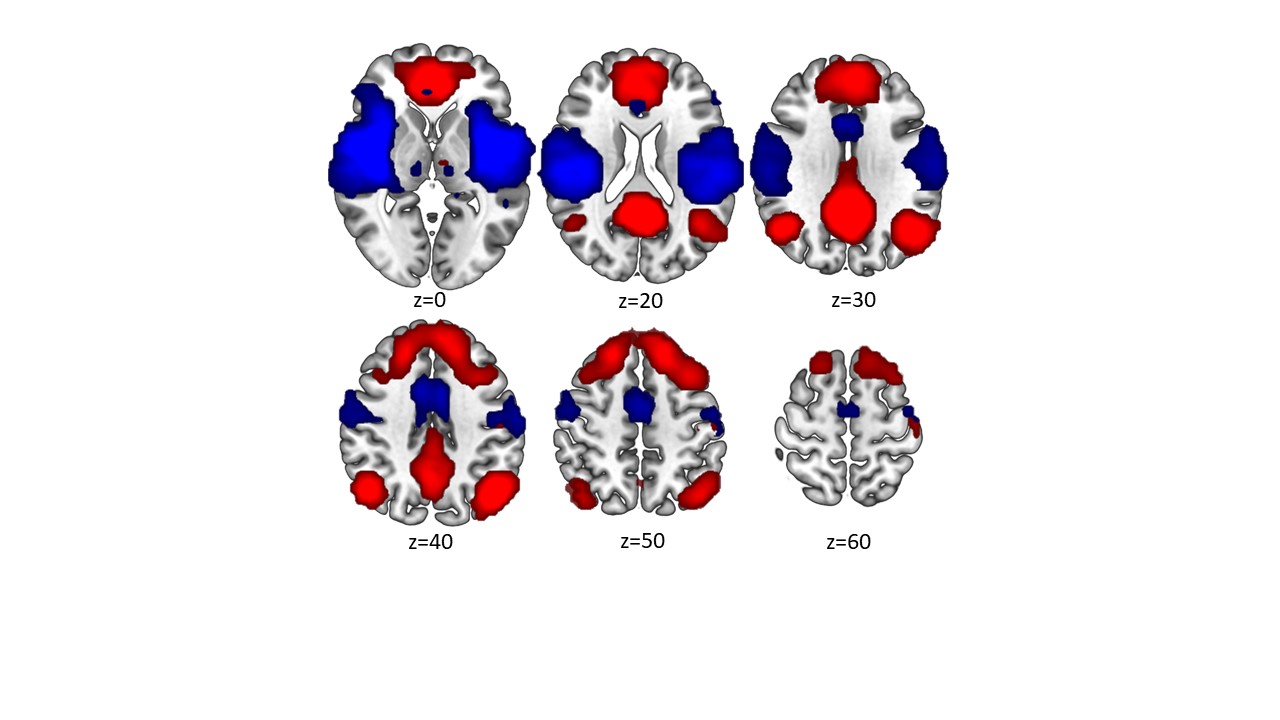


Figure S1. **Independent Component identified for testing.** Axial visualization of thresholded (z-score>3) ICs identified as Default mode Network (r-value=0.57), in red, and as Salience Network (r-value=0.40), in blue and chosen for statistical testing.
